# In-classroom physical activity breaks program among school children in Sri Lanka: study protocol for a randomized controlled trial

**DOI:** 10.3389/fpubh.2024.1360210

**Published:** 2024-04-22

**Authors:** D. L. I. H. K. Peiris, Yanping Duan, Corneel Vandelanotte, Wei Liang, Julien Steven Baker

**Affiliations:** ^1^Department of Sport, Physical Education and Health, Faculty of Social Sciences, Hong Kong Baptist University, Kowloon, Hong Kong SAR, China; ^2^Physical Activity Research Group, School of Health, Medical and Applied Sciences, Central Queensland University, Rockhampton, QLD, Australia; ^3^School of Physical Education, Shenzhen University, Shenzhen, China

**Keywords:** in-classroom physical activity breaks, behavior change wheel, academic achievement, health outcomes, fifth graders

## Abstract

**Background:**

The problem of sedentary behavior among primary school children is alarming, with numbers gradually increasing worldwide, including Sri Lanka. Physical activity interventions within classroom settings have been acknowledged as a critical strategy to increase students’ movement behaviors while enhancing their academic achievement and health. Yet, the busy curriculum and challenging educational demands encourage more sedentary classroom behavior. Hence, this study aims to develop and evaluate an in-classroom physical activity breaks (IcPAB) intervention among fifth graders in Sri Lanka.

**Methods:**

The study will adopt a randomized controlled trial (RCT), comprising an in-classroom physical activity breaks program group and a control group to evaluate the effects of IcPAB on academic achievement, movement behaviors and health outcomes. The intervention design is based on the capability (C), opportunity (O) and motivation (M) behavior (B) (COM-B) model. A least 198 fifth graders will be recruited from two schools in Uva province, Sri Lanka. The recruitment process will start in late 2022. Class teachers of the intervention group will implement 5-min activity breaks at least three times a day after completing a training session. The primary variables include mathematics and reading achievement. The secondary variables include physical activity levels, steps count, sedentary behavior, body mass index, aerobic fitness, and perceived stress. Data collection will be implemented at pre-test and post-test, respectively. Intervention fidelity and the process will also be evaluated.

**Discussion:**

The IcPAB is designed to prevent pure educational time loss by introducing curriculum-integrated short bouts of physical active breaks into the classroom routine. If the IcPAB is effective, it can (1) improve the mathematics and reading achievement of fifth-grade girls and boys, which is a significant factor determining the performance at the Grade Five National Scholarship Examination in Sri Lanka; (2) improve movement behaviors as well as physical and mental health outcomes among primary school students. Sequentially, the IcPAB will enrich school-based physical activity intervention approaches which can in turn bring academic and health benefits to primary school children in Sri Lanka.

**Trial registration:**

The first version of the trial was registered with the ISRCTN registry (Ref: ISRCTN52180050) on 20/07/2022.

## 1 Introduction

The effects of physical activity (PA) on academic achievement (1, 2), movement behaviors (3, 4) and health outcomes such as body mass index [BMI; (5, 6)], aerobic fitness (7, 8), and psychological health such as stress/ test anxiety levels (9, 10) among school children have been long established. However, reports including the elementary education sector highlighted that children should meet the recommended daily PA behavior levels, such as engaging in at least 60 min of moderate-to-vigorous physical activity [MVPA; (11)]. One primary factor influencing this figure is that children have long seated learning time in the classroom due to high academic curriculum demands in primary school (12, 13). As a result, sedentary behavior among primary school children has become alarming, with figures steadily increasing worldwide (14, 15).

A situation analysis conducted in 2016 showed that the learning time is less activity-based (16) in Sri Lanka. National surveillance data also indicated that 63 to 72% of students (age range = 6–12 years old) engaged in sedentary activities (17). Among the five grades in primary schools in Sri Lanka, the grade five curriculum is one of the most loaded and competitive in teaching and learning (18–20). The underlying reason is that grade five students are expected to sit for a national-level competitive scholarship examination in addition to their curriculum-related reviews at the term tests (18, 19, 21, 22). Therefore, the teachers and students are tense in preparing for the scholarship examination and surpassing the cutout marks while achieving the required competency levels for fifth graders (16, 19, 23, 24). This has increased the sedentary behavior with more traditional seated learning as the main objective of the teachers is to improve the students’ academic achievement. As a consequence, most of the fifth graders are identified to be physically inactive (17, 25–27), below recommended body mass index (BMI) percentiles (17, 25), aerobically unfit (18), and stressful (16, 19, 20, 23, 24, 28). Therefore, there is a need to target fifth graders in primary schools in Sri Lanka to prevent grade-related sedentary behavior and obtain health benefits and maximum academic performance.

It has been observed previously that PA breaks can improve the number of steps by 18% and minutes of MVPA by 26% during the school day (29, 30). Hence, there needs to be enablement to help manage the increased academic curriculum, and classroom settings are recommended as the best setting to achieve the benefits of PA (31–34). In the recent decade, few countries introduced the concept of in-classroom physical activity breaks (IcPAB) as a means of enhancing academic achievement (12), physical behaviors (35), and health outcomes (10, 36) by changing traditional seated learning into active learning among elementary school students. Still, more IcPAB initiatives are needed due to several limitations emphasized by a recent review based on the data from 1,538 primary school students (from 7 to 12 years old) in 10 studies (37):

(1) Not enough studies focused on introducing integrated PA into academic content through classroom-based interventions (37–40). (2) Most of those studies are introduced for populations from high-income Western countries (37, 39, 41) such as the USA, Australia, the Netherlands and Switzerland. The intervention effects based on gender are understudied. (3) Different durations were used in previous IcPAB initiatives, ranging from 10 min to 4,800 min (37), even though teachers prefer activity breaks that will take no more than 5 minutes (37), and the effectiveness of five-minute IcPAB interventions are understudied (37). (4) Previous studies demonstrated average methodological quality, with concerns around the randomization procedure, handling of missing data, outcome and process evaluation (37). This calls for future study protocols with a more robust methodological quality to avoid potential risks of bias in the IcPAB interventions, in addition to the well-explained outcome and process evaluation and fidelity methods. (5) Few studies used theoretical frameworks and evidence from related stakeholders such as teachers in designing IcPAB interventions. The review suggests (37) that robust PA interventions should be backed by well-established theories such as the capability (C), opportunity (O) and motivation (M) behavior (B) (COM-B) model (42) while considering the evidence-based opinions of the beneficiaries of the IcPAB in implementing an IcPAB program.

Therefore, there is a need for more IcPAB initiatives integrated into the curriculum across different geographic locations/ cultures (37) that require shorter bouts of time [i.e., around 5 minutes (12, 43)] with strong methodological quality (39, 44), outcome and process evaluation procedures and theoretical underpinnings (37, 39, 44). Also, the moderating effects of gender on the intervention should be further examined. Thus, by addressing the research limitations, this study aims to develop, implement, and evaluate a 12-week IcPAB program among grade five primary school children in Sri Lanka.

Depending on the aforementioned rationale, the specific research questions of this study include: (1) What is the impact of the IcPAB intervention on the primary outcome, academic achievement (mathematics and reading achievement) during the school day? (2) What is the impact of the IcPAB intervention on the secondary outcomes, movement behaviors (PA levels, steps count and sedentary behavior) and health outcomes (BMI, aerobic fitness, and perceived stress) during the school day? It is hypothesized that the IcPAB group will show improvement in all measures compared to the control group.

## 2 Methods

This study received ethical approval from the Ethics Review Committee of the University of Kelaniya, Sri Lanka (Ref: UOK/ERC/SS/2022/009) and Hong Kong Baptist University (Ref: SOSC-SPEH-2022-23_113). The trial was retrospectively registered with the ISRCTN registry (Ref: ISRCTN52180050). Methods are reported by adhering to the Consolidation Standards of Reporting Trials (CONSORT) guidelines and the Standard Protocol Items: Recommendations for Interventional Trials (SPIRIT) Statement throughout the study. The completed SPIRIT Checklist is added as a Supplementary material S1.

### 2.1 Study design and selection of subjects

A single-blinded parallel randomized controlled trial (RCT) will evaluate the intervention effects of IcPAB compared to a control group. Target participants will be the fifth graders in government primary schools. Based on the data shared by the Ministry of Health, Sri Lanka, the COVID-19 pandemic severity was slight in the Bandarawela Education Zone (city) in Badulla District. Therefore, the government schools of Bandarawela Education Zone were contacted to initiate the recruitment process in late 2022. The age range of the grade five students is 9 to 10 years old. The targeted population is exposed to 6 hours of regular classroom time (7.30 a.m. to 1.30 p.m.), including a 20-min lunch break according to the government primary school norms in Sri Lanka. Within the government primary school setting, the teacher in charge of a class should teach all subjects in the syllabus for the class, such as mother tongue, mathematics, religion, environment and English. Depending on the interests of the schools, the teachers will conduct additional classes after regular school time to prepare the students for the year-end national scholarship examination. Therefore, the study was designed to be implemented only during the standard classroom time for 12 weeks.

Based on GPower 3.1 software, through *a priori* power analysis, it was estimated to obtain at least 198 participants for both the intervention and control groups to expect an effect size of 0.21 [Cohen’s *f*, converted from *d*, (45, 46)] on academic achievement (mathematics) by providing a power of 0.8 (1-β) with an alpha of 0.05 to test the primary hypotheses of the study with an estimated 10% dropout rate [dropout rates were less than 10% in previous studies (10, 33, 47)] at the post-test/ follow-upstage.

Therefore, it is anticipated to recruit five to six classes (30 to 40 students per class) from two government schools to conduct the study to reach the required minimum sample size. Block randomization technique via MS Excel, will be used to randomize the sample classes by an assessor who is blinded to the intervention content and outcome assessment.

Permission was obtained first from the Bandarawela zonal education director to contact eligible government schools. For two schools to be eligible, grade five education must be offered, the students should not be exposed to similar interventions, and the principals must consent to join the research project. Once the principals grant the permission, the research team will contact the teachers in charge of the fifth-grade classes.

Teachers in charge of classes, parents/ guardians, and children at participating schools will be distributed with an informed consent form (Supplementary material S2) in plain language during the parents’ meeting days organized by the teachers. After obtaining the permissions, the principal investigator and the research assistants will visit the schools to collect data by giving prior notice to the teachers in charge of classes. The teachers will deliver the IcPAB. Therefore, all the students in the intervention classes will be exposed to IcPAB.

**Figure 1:**
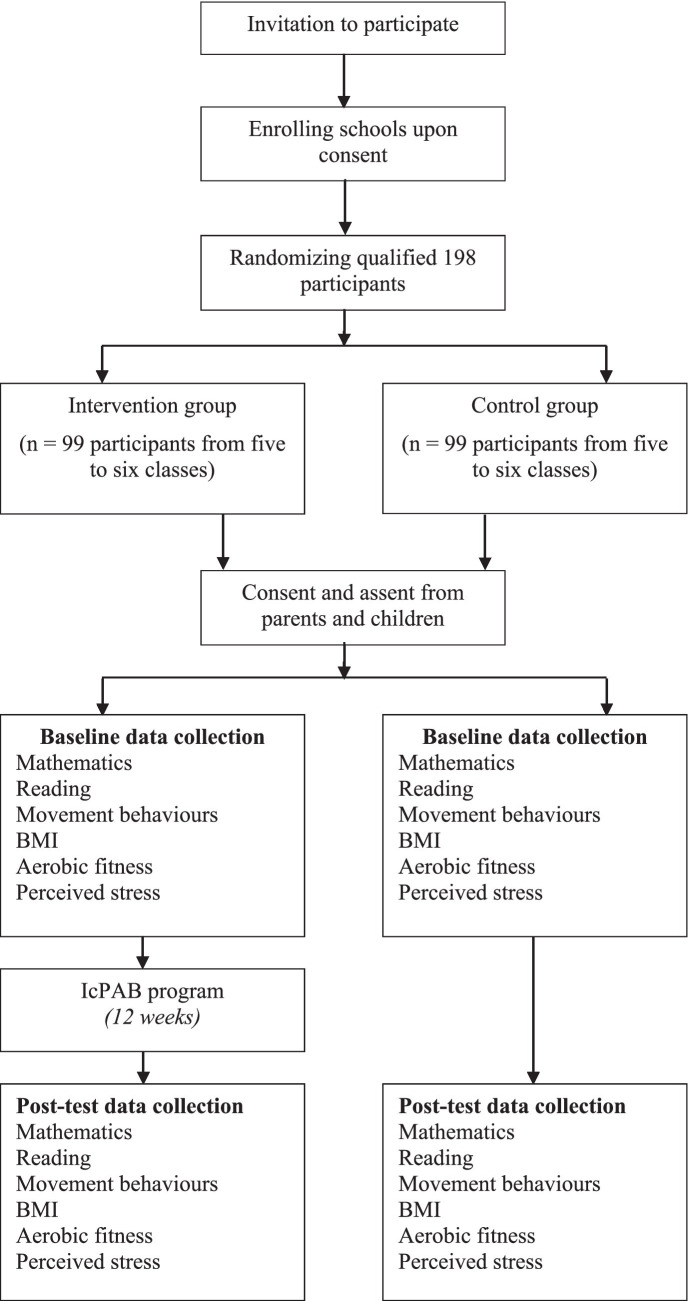
Flowchart of participants through the IcPAB program.

**Figure 2:**
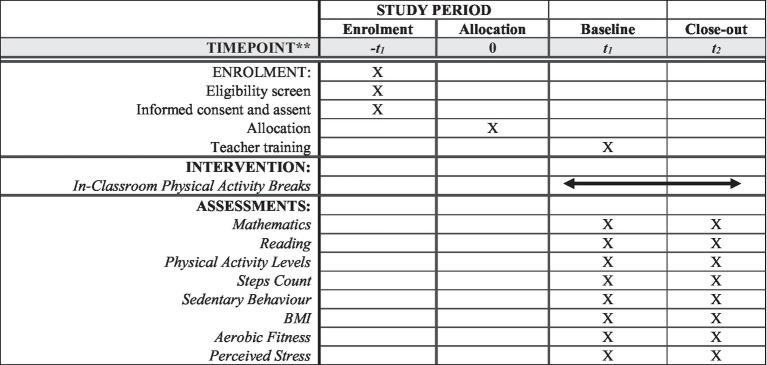
Intervention schedule for enrolment and assessments (SPIRIT Figure).

However, data will be collected only from the students meeting the consent requirements. The study will not include data from students with special health conditions and special education needs. An overview of the participant flow diagram (Figure 1) and the intervention schedule for enrolment and assessments [SPIRIT Figure (Figure 2)] are provided below.

### 2.2 Intervention

#### 2.2.1 Development of the intervention

Development of the intervention is facilitated through six steps. The conceptual design of mapping COM-B onto the suggested research work and the specification of each target behavior in developing and implementing the intervention is provided in Supplementary material S3.

Step 1: Systematically reviewed RCT-based IcPAB interventions for primary school students to identify the current practices at the global level. The review findings have been published elsewhere (37) by recommending the use of COM-B model as the theoretical basis for designing IcPAB interventions.

Step 2: Conducted semi-structured interviews with 21 primary school government teachers in Sri Lanka to analyse their perceptions on implementing IcPAB among Grade five students. The interview findings, which was thematically analyzed based on the COM-B model have been published elsewhere (20).

Based on the review (37) and the interview findings (20), the intervention was developed by embedding the Capability, Opportunity, Motivation-behavior (COM-B) model (20, 42, 48–50). The underlying theory of the COM-B model mentions that outcome behaviors must be understood in their context with consideration given to the individuals’ existing capability, opportunity and motivation to achieve these target behaviors (38, 42, 48, 51, 52). For example, it should be mentioned that the COM-B factors have been considered in IcPAB intervention and how they are addressed through the behavior change techniques (BCTs) (38, 53–55), which are defined as the active, observable, replicable, and irreducible components (42, 54) of the COM-B model.

Step 3: Identify the related COM-B that must be addressed to develop and implement the IcPAB. For example, as explicated in Table 1, it was identified that the class teachers’ dress code as a physical capability-related factor that should be concerned of when designing the IcPAB activities. Because teachers highlighted that they would have physiological limitations to demonstrate IcPAB as they are not wearing sports friendly wear at the classroom.

**Table 1:** Utilizing COM-B and BCTV1 taxonomy to develop and implement the intervention based on interview findings.

Based on interview findings: What encourages seated learning OR hinders implementing physically active teaching inside the classroom?	Which model of behavior sources (COM-B) is related to the interview finding?	What should be done to introduce IcPAB intervention by improving the identified behavior source model?	What behavior change techniques from BCTTv1 should be applied to activate the intervention functions and policy categories?
Physiological limits due to the age and dress code	Physical Capabilities	Provide examples from the field of education of how IcPAB can be used regardless of the physiological barriers.	4.2 Information about antecedents 5.3. Information about social and environmental consequences 9.1 Credible source 15.1 Verbal persuasion about capability, i.e., *Provide credible written materials and videos of how previous IcPAB practices are conducted despite teacher age and dress; tell teachers individually that they can successfully conduct IcPAB frequently*
		Convince the teachers that the IcPAB can be implemented without changing into a sporty dress.	6.1 Demonstration of the behavior 15.1 Verbal persuasion about capability, i.e., *Show the teachers in a real class situation how IcPAB can be done with the dress code for lady teachers (Saree); tell teachers individually that they can successfully conduct IcPAB frequently.*
Lack of psychological confidence to implement physical activities other than the specified activities in the teachers’ guide.	Psychological Capabilities	Provide easy-to-understand written and visual manuals on conducting IcPAB.	4.2 Information about antecedents 9.1 Credible source, i.e., *Provide a manual specifically designed for Sri Lankan teachers with pictures and videos.*
		Provision of regular training and feedback on IcPAB implementation.	4.1 Shaping knowledge 6.1 Demonstration of the behavior 15.1 Verbal persuasion about capability, i.e., *Hold special training sessions for teachers, advise and agree on how to perform IcPAB; Implement IcPAB with teachers/ provide assisted delivery; provide weekly feedback to the teacher and tell them they can successfully conduct IcPAB frequently.*
Not receiving external enablement to conduct classroom-based physical activities.	Social Opportunities	Request school-level policy decisions to implement IcPAB.	1.2 Problem-solving, i.e., *Hold meetings and request permission from section heads to implement IcPAB as a novel teaching strategy and to test its effectiveness on academic achievement.*
		Set goals to achieve a minimum number of IcPAB daily.	1.1 Goal setting (behavior) i.e.*, Set a goal to implement 5-min IcPAB at least thrice daily.*
		Regular follow-up and monitoring of the fidelity.	7.1. Prompts/ cues 2.2 Feedback on behavior, i.e., *Send daily WhatsApp messages to teachers to implement IcPAB at least three times; monitor the way the teachers conduct IcPAB and provide informative, evaluative feedback.*
Most classrooms are crowded and need more space to conduct physical activities.	Physical Opportunities	Convince the teachers that the IcPAB can be implemented in a small space just by standing in front of the desk.	6.1 Demonstration of the behavior 15.1 Verbal persuasion about capability, i.e.*, Show the teachers in a real class situation how IcPAB can be done just by standing behind the desk; tell teachers individually that they can successfully conduct IcPAB frequently.*
Not receiving updated information on the risks of prolonged sitting	Automatic Motivation	Provide examples of the risks of prolonged sitting and the benefits of doing IcPAB.	5.1 Information about health consequences 5.3. Information about social and environmental consequences 9.1 Credible source, i.e., *Provide credible written materials and videos on the risks of prolonged seating and how such risks can be minimized.*
Lack of strong will to break sitting time and belief in the benefits of engaging in PA inside a classroom	Reflective Motivation	Convince the teachers with evidence that the IcPAB are integrated into the curriculum and that the activities help enhance academic performance.	4.2 Information about antecedents 5.3. Information about social and environmental consequences 9.1 Credible source, i.e., *Provide credible evidence from previous research findings on how IcPAB helped enhance student performance in various subjects.*

Step 4: Applied the BCTs from the BCTv1 taxonomy to mitigate the identified behavioral issue (Table 1) to enhance academic achievement, movement behaviors and the health outcomes of the target group. For example, it was identified that the teachers’ doubts on physical capability factors such as dress code-related physiological limits can be mitigated by providing credible written materials and videos of how previous IcPAB practices are conducted despite teacher age and dress (BCT 9.1) and telling teachers individually that they can successfully conduct IcPAB frequently (BCT 15.1). Further details are provided in 2.2.2 below.

Step 5: Designed IcPAB activities and facilitator packs with local educators based on the evidence collected from the review and the interviews. For example, a manual, logbooks, timers, WhatsApp messaging group and 20 IcPAB cards were prepared as intervention materials. The details of IcPAB cards will be illustrated in 2.2.2 below.

Step 6: Tested the IcPAB cards in a pilot test with two teachers from two classes in one school. This school will not be involved in the main intervention. The aim of this pilot is to test the ability of the teachers to follow the instructions provided on IcPAB cards and to observe the students’ reaction toward the activity breaks.

Step 7: As elaborated in 2.2.2, conduct a training for the intervention class teachers to be familiar with the IcPAB program.

The first six steps were completed, and the Step 7 will be facilitated in future.

#### 2.2.2 Delivery of the IcPAB in the intervention group

The IcPAB program will introduce 20 cards among all intervention classes. The interviews (20) conducted during the intervention development stage (Step 2 in section 2.2.1) explored that activities which take around 5 minutes would be feasible to implement in class due to high density of teaching content in each regular class. Also, the previous evidence assured IcPAB, which requires less than 5 min to provide positive intervention effects (12, 56). Therefore, the teachers will be requested to implement at least five-minute PA breaks three times daily. Each activity should be implemented for at least 5 minutes.

The IcPAB cards can be incorporated into the mathematics and language reading lessons. All the cards are designed for teachers to teach mathematics and language lessons using physically active teaching methods while reducing the fifth graders’ school day seated learning time without interrupting academic teaching time. The nature of the activities will also assist students to improve their aerobic fitness and enjoy learning by forgetting any stressors. Each card has a picture resembling a particular IcPAB. The back of the card has written instructions on performing the activities in addition to the instructions provided in the IcPAB manual. The manual was designed for Sri Lankan teachers with pictures by merging the activities with the fifth-grade curriculum with PA.

The first card is designed to implement a breathing exercise as students require stress relief. Hence, the teachers can choose either the first IcPAB card with another card as the first activity of each day or another activity card. Even if the teachers decide not to use the IcPAB card one, they are given instructions on each card to do a little breathing before starting any activity if deemed necessary. Teachers are reminded on each card to use encouraging words (such as very good) to finish the activity and refer to the lesson.

For example, as provided in Supplementary material S4, the first card (IcPAB card ID: IcPAB 3) is designed to help the students with arithmetic skills. If a teacher wants the students to relax by doing a breathing exercise, the students will be instructed to stand comfortably and inhale- exhale three times by raising up and down on the toes while moving the hands up and down in the same rhythm. Then, the students will solve a multiplication problem given by the teacher (if the problem is more complicated, ask students to use a piece of paper while standing) and jump on the spot equal to the obtained answer. Next, the teacher will repeatedly solve the question with the students and jump on the place. The students will be given a verbal appreciation with or without a clap.

The teacher training (BCT 6.1) will be the primary enablement of the teachers to engage in the intervention. Teachers involved in the intervention will receive one-hour training 1 week before the commencement of the IcPAB intervention. The principal investigator will conduct the training session in a classroom after the regular school time upon the teachers’ availability. Teachers will receive intervention materials such as the IcPAB manual, IcPAB cards, timers, and the logbook. Intervention materials are written in the local language (Sinhala). However, English terms are used in the materials when deemed necessary.

By addressing the findings from the interviews (20), the training session with the application of the BCT taxonomy (i.e., BCT 4.1; 4.2; 5.1; 5.3; 9.1) will cover (1) the importance and benefits of IcPAB by referring to previous research findings, (2) rationale of introducing the current IcPAB activities, (3) recording of the logbooks (4) demonstration of IcPAB and (5) questions and answer round. i.e., at training, teachers will be given credible written materials and videos of how previous IcPAB practices are conducted despite teachers’ age and dress (BCT 4.2; BCT 5.3 and BCT 9.1). The teachers will be convinced that the IcPAB can be implemented without changing into a sporty dress by demonstrating some activities during training in addition to the assisted delivery in the classroom (BCT 6.1). Also, teachers will be convinced about the risks of prolonged seating and how such risks can be minimized through IcPAB can be used (BCT 5.1; BCT 5.3 with research evidence (BCT 9.1) in addition to how IcPAB helped enhance student performance in various subjects (BCT 4.2 and 5.3) in other countries.

The principal investigator will deliver and observe the IcPAB program during the first two weeks of intervention to ensure adherence to the intervention protocol by following a previous practice (12). Therefore, extensive demonstrations of the active break activities will not be provided during the training. i.e., Following a previous intervention practice called assisted delivery (12), the principal investigator will deliver the intervention activities together with classroom teachers during the first week of the intervention. During the second week, the principal investigator will observe teachers doing the activities and provide support if needed. This assisted delivery method (BCT 6.1, BCT 4.1) during the first week of the intervention and the IcPAB manual (BCT 9.1) will enable the teacher to engage in the intervention activities continuously. Teachers are given the opportunity to replicate/ modify the PA breaks. If this is done, the teachers will record their actions in the logbook while noting the detailed information on the last pages of the IcPAB manual. Replicated/ modified activities will be given feedback by the principal investigator.

Additionally, prompts (BCT 7.1) such as WhatsApp messages and interactive discussions (BCT 15.1) with the teachers will be used as techniques to implement the intervention to remind and persuade them to carry out the IcPAB each week at least to meet the minimum required dose (BCT 1.1). Furthermore, should the teachers face any difficulty, they will be assisted by the principal investigator or the research assistants to manage the issues (BCT 1.1) while providing informative, evaluative feedback (BCT 2.2) about their progress with the intervention activities. The feedback will also include the satisfaction of students.

However, should the children or the classroom teachers not want to implement intervention activities, they can do so without any reason. No participants will be advantaged or disadvantaged in any way by doing so. Parents, children, and teachers will be reassured that they can withdraw their permission anytime during this project without penalty. No foreseeable added risk was identified above the risks of everyday life. In addition, implementing IcPAB would not harm the students’ physical and emotional health or the pure educational time as the teachers will be given complete autonomy to choose the most appropriate time to carry on IcPAB.

#### 2.2.3 Control group

The classes that will be randomized in relation to the control group will not receive the IcPAB within 12 weeks of the intervention. However, those schools will be given all the resources to implement IcPAB activities once the post-test data collection is fulfilled. During the intervention period, the control group’s teachers will be contacted once weekly through WhatsApp phone calls by the principal investigator and twice a week physically by the principal investigator/ research assistants. Correspondence will be maintained to obtain information about their lesson delivery patterns to ensure that the control group did not receive interventions to change their normal study modes.

### 2.3 Measurements

Synthesis of primary (academic achievement) and secondary outcomes (movement behaviors and health outcomes) measurements in this study are shown in Supplementary material S5. All measurement outcomes will be compared among the control group. Data collection for measuring outcomes will be conducted by adhering to the Helsinki Declaration, national and school-level safety protocols and COVID-19 prevention guidelines.

#### 2.3.1 Primary outcomes

Based on the interviews conducted with 21 teachers from the nine provinces of Sri Lanka (interview findings were published elsewhere; (20)), mathematics and reading performance were identified as the most important subjects for the students to perform well at the Grade Five national level scholarship examination. In the Sri Lankan and international contexts, it is evident that mathematics and reading performance are the key pillars of elementary education’s academic achievement (10, 12, 32, 33, 57).

##### 2.3.1.1 Mathematics achievement

Mathematics achievement will be evaluated through a curriculum-based standardized test designed by the teaching officers experienced in Grade five mathematics performance-related evaluation. This test will consist of 60 questions to assess the expected performance of a given term. Students will complete the test within 45 min. The principal investigator and the classroom teacher will collaboratively administer the test.

##### 2.3.1.2 Reading achievement

Reading achievement will be evaluated through a standardized reading test specific to the Sri Lankan Grade Five curriculum. Three grade five teachers have chosen two paragraphs to be used at the baseline and at the end of the intervention to evaluate the students’ reading achievement. The paragraphs include around 200 to 250 words; each student will read the sections for 2 minutes. Reading performance will be evaluated by a teacher who is not the student’s classroom teacher (yet a classroom teacher of the same school’s parallel grade) under the principal investigator’s distant observation.

#### 2.3.2 Secondary outcomes

Movement behaviors such as PA levels, steps count, and sedentary behavior within regular school hours will be evaluated. Health outcomes such as BMI, aerobic fitness, and perceived stress will be assessed as other secondary outcomes.

##### 2.3.2.1 Movement behaviors

Objective data for the light physical activity (LPA), moderate physical activity (MPA), vigorous physical activity (VPA), MVPA, steps count, and sedentary behavior will be measured during regular school hours using waist-worn accelerometers (GT3-X triaxial model, ActiGraph LLC, Pensacola, Fla., USA). Accelerometer data will be collected for a week at baseline and the post-test (week 13).

The accelerometers will be distributed by the teachers to the students randomly on the first school day and collected back on the fifth school day of the week. The research team will demonstrate to the teachers and the students how the accelerometers should be worn on the first day. However, due to the limited number of accelerometers, this study will use a randomly selected subsample (n = 47) to collect data to measure all the movement behaviors following previous research practice (12, 34, 58, 59). After accelerometers are randomly distributed to the students on the first day, the teachers will prepare a list assigning each student to a specific numbered accelerometer to ensure that the same child wears the same device every day during all the data collection stages. Then, the research team will receive another version of the same list where the students’ anonymous identification number is related to the accelerometer. The classroom teacher will also record absent students assigned with an accelerometer, and a researcher assistant will verify the data during the school visits.

Based on previous research practices (12, 60), only the accelerometer data, which will be identified for wearing more than five school hours on at least one school day, will be included in the analysis for intervention effects (12, 60). Following the standard practices in the studies involving children, non-wear time will be defined as 20 min of consecutive zeros (12, 31, 61). Freedson cut points will be used to classify movement behavior intensities based on the data collected in 15-s epochs (12, 62). Due to the focus on in-school PA, a longer wear time is not deemed necessary for the current study, as recommended by recent scholars (63). To confirm in-school wear-time for valid days, a further visual check of each accelerometer profile will be undertaken (63). Therefore, research assistants will randomly visit the schools during the data collection week to ensure that the students wear the same device accurately during school hours.

##### 2.3.2.2 Health outcomes (BMI, aerobic fitness, perceived stress)

Students’ weight in kilograms to the nearest 0.1 kg (27, 63, 64) and height in centimeters to the nearest 0.1 cm (27, 63, 64) will be recorded using a standard stadiometer and a weighing scale. Body weight in kilograms will be divided by height in meters squared to measure the BMI (27, 63, 64). Students’ age and gender-specified BMI categories will be identified using the calculator introduced by the Ministry of Health in Sri Lanka (65).

The multistage shuttle run/ beep test [66] will be used to measure aerobic fitness, proven highly reliable and valid among school children (32, 66). Results from the test will be used to calculate VO_2_ max using the equation proposed by previous studies (67, 68). Students will be asked to run back and forth on a 20 m course as instructed by a sound signal emitted from a pre-recorded tape, ensuring they touch the 20 m line with their foot (32). The sound signal frequency increases by 0.5 km/h every minute, indicating the next stage (level), starting with a speed of 8.5 km/h. The test ends when participants fail to reach the line before the signal.

Perceived stress will be measured using a translated Sinhala version of the Perceived Stress Questionnaire 8–11 (PSQ8-11 (67)). PSQ8-11 will measure two subscales: perceived psychological stress (nine questions) and physiological stress (10 questions). This 19-item questionnaire requires the students to recall their feelings from the previous week. The students will self-rate their responses on a four-point Likert scale (1 = never, 2 = sometimes, 3 = often, 4 = very often). A higher score on the questionnaire will indicate greater perceived stress (67).

### 2.4 Fidelity of the intervention

The primary source for assessing the fidelity will be the logbooks of the teachers. An example of a log sheet can be found in Supplementary material S6. Teachers will indicate how many IcPAB they implemented daily throughout the intervention period. Teachers will be reminded every day via WhatsApp messages to carry out IcPAB, and a research team member will visit the schools once a week to check if the logbooks are duly filled. The personal visits to the intervention schools will also minimize issues with time management and PA breaks implementation from the teachers’ side. In addition, accelerometer data from the post-test will be compared with the responses obtained through teacher and student interviews at the post-test.

### 2.5 Process evaluation

During the intervention, the principal investigator will receive teacher feedback and give subjective evaluations on how the teachers implement the IcPAB. At the same time, the research team will obtain verbal feedback once a week from the students on IcPAB’s ability to provide fun and engagement using two close-ended questions: (1) Did you enjoy the activities today? (2) Was it easy for you to follow the instructions and do the activities today? This ongoing feedback-receiving process will be used to overcome any identified or foreseen challenges to implement the IcPAB program (31, 43).

In addition, after the 12-week RCT intervention program, a five-point Likert-scale questionnaire will be distributed among the intervention group’s teachers and students to analyse process evaluation outcomes (Supplementary material S7). The teachers’ questionnaire consists of eight items, while the students’ questionnaire consists of nine items. Both the questionnaires were adopted from previous research work (56, 69).

#### 2.5.1 Sustainability evaluation

As a sustainability strategy to track the program for future adoptions all the teacher facilitators and randomly selected student groups will be contacted. The aim is to explore the perceptions of teachers and students in attending and implementing in-classroom PA breaks program through a semi structured interview study based on the capability, opportunity, and motivation behaviors model. The interview guide is available in Supplementary material S8.

### 2.6 Data analysis

Data will be statistically analyzed using IBM SPSS software version 28. A randomization check will be performed using independent t-tests (for continuous outcomes) and chi-square (for categorical variables) tests (70). Descriptive statistics such as mean, standard deviation, and percentages will be used to describe the baseline characteristics of the sample and the attrition rates. Primary analysis will be handled with an intention-to-treat modified (m-ITT) approach (71). Missing values analysis will be conducted to observe whether the data are missing completely at random (MCAR). Missing data values will be handled using the multiple imputation method with chained equations.

To test the effectiveness of the IcPAB, generalized linear mixed models (GLMM) will be used by linking to the dependent variables at the individual level (students) and group level (intervention vs. control) with time (pre to post-test) (12, 72, 73). A random intercept will be used to account for the repeated measures of the subjects (35). To test the moderation effects by gender, an interaction test will be conducted via GLMM by setting group-by-time-by-gender as the moderator (35). A 5% level (two-tailed) will be used as the statistical significance cut-off point (74). All the intervention effects will be reported based on the type III tests of fixed effects of GLMM. The estimates of the impact sizes will be written based on the estimated coefficients (*β*) with the 95% confidence intervals/ odds ratio for those estimates based on previous intervention effect report practices for IcPAB (31, 63) in addition to the mean changes in the intervention and control groups. The odds ratio (OR), where OR = 1.68, 3.47, and 6.71 are equivalent to Cohen’s *d* = 0.2 (small), 0.5 (medium), and 0.8 (large), will be used for the interpretations, respectively, (75). Furthermore, a sensitivity analysis to the m-ITT results will be facilitated by considering all the subjects who complete all the baseline and post-test measurements for all the primary and secondary outcomes to ensure the robustness of the primary data assessment and strengthen the conclusions and credibility of the study’s findings.

## 3 Discussion

The primary aim of this RCT is to investigate the effects of the IcPAB program on the mathematics and reading achievement of fifth graders in Sri Lanka. Secondarily, the intervention effects of the LPA, MPA, VPA, MVPA, steps count, and sedentary behavior will be evaluated in addition to the health outcomes such as BMI, aerobic fitness, and perceived stress.

This intervention has addressed several recommendations and limitations pointed out by previous studies: (1) To the best of the authors’ knowledge, this is the first IcPAB intervention, which will be implemented among governmental primary school girls and boys in Sri Lanka by integrating mathematics and reading curriculum components into the IcPAB content. The intervention will analyse its moderation effects by gender. Thus, this intervention’s findings will fill an existing population gap and add new knowledge to academia (39, 41). (2) It is reported that the IcPAB, which requires a more prolonged duration per activity, are less feasible (12, 39, 76). At the same time, teachers prefer shorter bouts of IcPAB, at least at most 5 minutes (20, 37). This intervention will provide more evidence of the effects of using five-minute curriculum-integrated PA breaks.

(3) The intervention uses a RCT with a parallel single-masked design, ensuring a low bias risk to its methodological quality (39, 41, 44). Also, the protocol contains a precise data analysis plan for handling missing data and examining the data with a sensitivity analysis. (4) Intervention fidelity and process evaluation mechanisms will be systematically followed and reported to ensure the feasibility of the RCT in the ‘real-world’ context (12, 39). As for the primary outcome evaluations, curriculum-based measurements are designed. Curriculum-based measures were recommended by the previous researchers due to its sensitivity toward small changes (12, 39) and the ability to be administered frequently (12, 39).

In addition, this intervention will use accelerometer data to analyse the effects of movement behaviors. This is a strength of the IcPAB intervention, as objective measures to record PA levels, steps count, and sedentary behavior are strongly recommended (12, 44). Furthermore, the effects of IcPAB on BMI, aerobic fitness and stress levels are under-researched among preadolescents (67, 77–81). In particular, higher stress levels among fifth graders in Sri Lanka are a critical issue (18, 28, 81). Global evidence also suggests that there are few efforts to be found in analyzing the effects of academic load on stress levels among preadolescents and adolescents (67, 77–81). (5) The intervention materials were developed with fifth-grade teachers in Sri Lanka by analyzing the current capabilities, opportunities, and motivations of the prospective IcPAB implementors (20). Therefore, this intervention is equipped with a theoretical foundation, COM-B model (38, 39, 82). Thus, the IcPAB program will address these facets, a significant strength of this RCT.

### 3.1 Limitations

Despite the strengths mentioned above, it is possible to indirectly affect the fidelity of the intervention due to the ongoing economic crisis and post-COVID-19 pandemic in Sri Lanka (83, 84). For example, the sudden closure of schools for a few days will reduce the number of days the IcPAB can be implemented weekly (83–85). Furthermore, participants were limited to the Uva Province in Sri Lanka. This will cause a representation bias in the data (86). The classes are randomized into intervention and control groups. This may cause potential contamination effects. Therefore, use of a cluster-RCT design is recommended to in the future studies similar to a previous study (31). There may be a potential for bias as the same teacher who is delivering the IcPAB records the fidelity of the intervention (12) even though the research team plans to conduct weekly observations of the frequency of conducting IcPAB. Also, the results may be limited by poor protocol compliance if few classes are included in the study as “one teacher could skew the results” (31).

Even though, this study was designed based on COM-B model to address the hindrances to implement classroom-based PA, effect of the factors such as participants’ dietary habits (87), challenges with school initiatives (88), children’s preferences for outdoor activities (89) will not be measured throughout the proposed RCT.

### 3.2 Significance of the study

Using the COM-B model as a theoretical underpinning and behavior change technique on teacher facilitators will provide more opportunities for children to be active at school. Active children are identified to improve their academic performance, physical behaviors, and health. Therefore, this study could improve the mathematics and reading achievement of fifth-grade girls and boys, which is a significant factor determining the performance at the Grade Five National Scholarship Examination in Sri Lanka. At the same time, the IcPAB program will help improve healthy behaviors and health, including emotional health, among primary school students. Furthermore, the IcPAB program was designed to prevent pure educational time loss by introducing curriculum-integrated short bouts of physically active breaks into the classroom routine. Therefore, future findings of this study will be significant in providing positive effects of IcPAB on primary school children as an RCT that addresses several limitations of previously implanted IcPAB programs.

## Ethics statement

The studies involving humans were approved by the Ethics Review Committee of the University of Kelaniya, Sri Lanka (Ref: UOK/ERC/SS/2022/009) and Hong Kong Baptist University (Ref: SOSCSPEH-2022-23_113). The studies will be conducted in accordance with the local legislation and institutional requirements. Written informed consent will be obtained from the participants' legal guardians/next of kin prior to participation in the study.

## Data availability statement

Analysed data from the intervention will be published upon the completion of the intervention. Any data that would disclose the participants’ personnel information will not be made public.

## Author contributions

DP: Conceptualization, Data curation, Formal analysis, Funding acquisition, Investigation, Methodology, Project administration, Resources, Validation, Visualization, Writing – original draft, Writing – review & editing. YD: Conceptualization, Methodology, Project administration, Supervision, Validation, Writing – review & editing. CV: Conceptualization, Methodology, Resources, Supervision, Writing – review & editing. WL: Conceptualization, Methodology, Project administration, Supervision, Writing – review & editing. JB: Conceptualization, Methodology, Supervision, Writing – review & editing.
