# Supplementary material for: In-classroom physical activity breaks program among school children in Sri Lanka: study protocol for a randomized controlled trial
Source: Front Public Health. 2024 Apr 22;12:1360210. doi: 10.3389/fpubh.2024.1360210 (PMC11070516; doi:10.3389/fpubh.2024.1360210)
Supplement: Supplementary file 2 [file Data_Sheet_2.PDF]

(Informed Consent Form – Parents)

Dear Parents,

This is with regard to what I have explained to you at the parents' meeting today, about my research on improving your child's academic achievement, healthy behaviours and health outcomes through physical activity breaks. If you agree or disagree with the following statements, please initial the relevant box with a " ✔ ". Thank you very much for your time and support.

D.L.I.H.K. PEIRIS,
Principal Investigator of the research,
Hong Kong Baptist University.

Statement	Agree	Disagree
I confirm that I understood the opportunity to listen to the reasons, procedure, benefits, and safety concerns of conducting this research.		
I have read the information sheet, which provided a written explanation of this research activity, which involves my child/ children.		
I had the opportunity to ask for more information, and questions about this research, and I received satisfactory answers for my inquires by the researcher.		
I understand that information of my child/children such as test marks for mathematics and reading, physical activity and fitness-related data, and perceived stress scores will be used by the researcher to evaluate the effectiveness of physical activity breaks on my child.		
I give permission to the researcher to collect those data from my child under an anonymous identifier.		
I agree to audio/ video record the classroom activities of my child, use of anonymised quotes for the research when deemed necessary to the researcher.		
I understand that my child's/ children's participation in the research activity is completely voluntary, and non-participation will not create any disadvantage to him/her/them.		
I understand that any data collection will be carried under the supervision and observation of the school, adhering to all health and safety protocols.		
I understand that all the data collected in this research, will not reveal my child's privacy and stored in a password protected, encrypted computer, which is only accessible to the researcher.		
I know that I have the authority to ask the researcher to not to collect any data from my child/ children and to receive clarifications from the researcher at any time point during the research activity period.		
I hereby, give my consent to the researcher to involve my child/ children in the physical activity breaks project and to collect his/her/their data.		
Parent's/ Guardian's name:		
Signature:		
Date:		

Annexe – II (Informed Consent Form – Teachers)

Dear Teacher,

This is with regard to what I have explained to you at the teacher briefings and parents' meeting today, about my research on improving your students' academic achievement, healthy behaviours and health outcomes through physical activity breaks. If you agree or disagree with the following statements, please initial the relevant box with a "✔". Thank you very much for your time and support.

D.L.I.H.K. PEIRIS,
Principal Investigator of the research,
Hong Kong Baptist University.

Statement	Agree	Disagree
I confirm that I understood the opportunity to listen to the reasons, procedure, benefits for students, and safety concerns of conducting this research.		
I have read the information sheet, which provided a written explanation of this research activity, which involves my students.		
I had the opportunity to ask for more information, and questions about this research, and I received satisfactory answers for my inquires by the researcher.		
I understand that information of my students such as test marks for mathematics and reading, physical activity and fitness-related data, and perceived stress scores will be used by the researcher to evaluate the effectiveness of physical activity breaks on the students.		
I give permission to the researcher to collect those data from the students under an anonymous identifier, assigned by me after receiving parents' consent.		
I agree to audio/ video record the classroom activities, use of anonymised quotes for the research when deemed necessary to the researcher.		
I understand that the students' participation in the research activity is completely voluntary, and non-participation will not create any disadvantage to them.		
I understand that any data collection will be carried under my and schools' supervision and observation, adhering to all health and safety protocols.		
I understand that all the data collected in this research, will not reveal students' privacy and stored in a password protected, encrypted computer, which is only accessible to the researcher.		
I understand that implementing activity breaks in the classroom are completely voluntary, and I can give-up my participation at any time.		
I know that I have the authority to ask the researcher to not to collect any data from the students' and to receive clarifications from the researcher at any time point during the research activity period.		
I hereby, give my consent to the researcher to involve my classroom and the students in the physical activity breaks project and to collect necessary data.		
Teacher's name:		
Signature:		
Date:		

Annexe – II (Informed Consent Form – Grade V Students)

Dear Daughter/ Son,

This is with regard to what I have explained to you today at the classroom, about my efforts on improving your academic achievement, healthy behaviours and health outcomes through physical activity breaks. If you agree or disagree with the following statements, please initial the relevant box with a " ✔ ". Thank you very much for your time and support.

D.L.I.H.K. PEIRIS,
Principal Investigator of the research,
Hong Kong Baptist University.

Statement	Yes	No
I confirm that I understood the opportunity to listen to the reasons, procedure, benefits, and safety concerns of implementing physical activities during the classroom time.		
I have read the information sheet, which provided a written explanation of this research activity, which involves me and my classroom.		
I had the opportunity to ask for more information, and questions about the activities that will be implemented in the classroom, and I received satisfactory answers for my inquires by Ms. Peiris.		
I understand that my test marks for mathematics and reading, physical activity and fitness-related data, and perceived stress scores will be used by Ms. Peiris to evaluate the effectiveness of physical activity breaks implemented in the classroom.		
I give permission to Ms. Peiris to collect those data from me under an anonymous identifier, assigned by my teacher after receiving my parents' consent.		
I agree to audio/ video record our classroom activities, use of anonymised quotes for Ms. Peiris's research when deemed necessary to her.		
I understand that my participation in the research activity is completely voluntary, and non-participation will not create any disadvantage to me.		
I understand that any data collection will be carried under my teacher's and schools' supervision and observation, adhering to all health and safety protocols.		
I understand that all the data collected by Ms. Peiris, will not reveal my privacy and stored in a password protected, encrypted computer, which is only accessible to Ms. Peiris.		
I understand that implementing activity breaks in the classroom are completely voluntary, and I can give-up doing activities at any time.		
I know that I have the authority to ask Ms. Peiris to not to collect any data from me and to receive clarifications from her at any time point during her research activity period.		
I hereby, give my consent to Ms. Peiris to involve me at the classroom in the physical activity breaks project and to collect necessary data.		
Your name:		
Signature:		
Date:		
