# Supplementary material for: In-classroom physical activity breaks program among school children in Sri Lanka: study protocol for a randomized controlled trial
Source: Front Public Health. 2024 Apr 22;12:1360210. doi: 10.3389/fpubh.2024.1360210 (PMC11070516; doi:10.3389/fpubh.2024.1360210)
Supplement: Supplementary file 3 [file Data_Sheet_3.PDF]

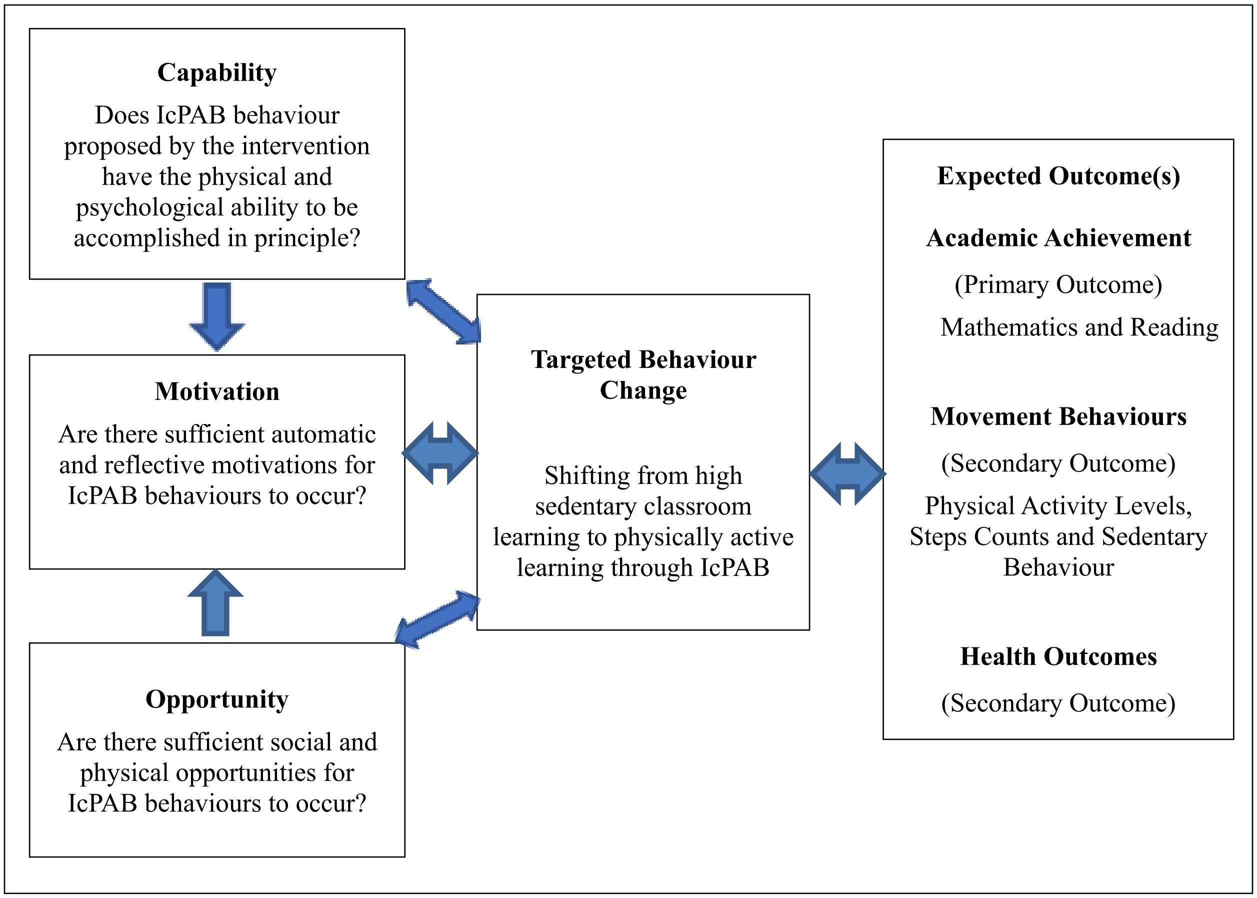


*Supplementary Figure. Mapping the COM-B Model onto intervention concept (Adapted* [1,2]*)*


1. Brierley ML, Smith LR, Bailey DP, Every SA, Staines TA, Chater AM. Perceived influences on reducing prolonged sitting in police staff: a qualitative investigation using the Theoretical Domains Framework and COM-B model. BMC Public Health [Internet]. 2021 Dec 19 [cited 2022 Aug 29];21(1):2126. Available from: https://link.springer.com/articles/10.1186/s12889-021-12019-6

2. Ojo SO, Bailey DP, Brierley ML, Hewson DJ, Chater AM. Breaking barriers: using the behavior change wheel to develop a tailored intervention to overcome workplace inhibitors to breaking up sitting time. BMC Public Health [Internet]. 2019 Dec 16 [cited 2021 Nov 2];19(1):1126. Available from: https://bmcpublichealth.biomedcentral.com/articles/10.1186/s12889-019-7468-8

*Supplementary Table. Specific procedure of development and implementation of the ICPAB intervention*

| Targets | Who | Where | When | How often | With/From/To whom |
|---|---|---|---|---|---|
| Obtain information about the capability, the desire of the facilitators, and opportunities to implement an IcPAB intervention in SL | Research Team | At the field locations where interviews are conducted | During pre-intervention stage | Once from each key informant | Key informants such as teachers, section heads and principals |
| Develop IcPAB | | Workstations | | Number of modifications until an agreement was made | Teachers |
| Share evidence-based information on the benefits of IcPAB | | At schools | | Once | Principals |
| Enrolling and randomizing the schools | | Workstations | | | An independent randomizer |
| Obtaining consents | | At school | | | Parents/ guardians |
| Share evidence-based information on the benefits of IcPAB | | At teacher training location | | | IcPAB facilitators (Teachers) |
| | | At IcPAB intervention venue | | When the need arises | |
| Delivering intervention manual | | At teacher training location | | Once | |
| IcPAB training | | | | | |
| Obtain baseline measurements | | At school | During the intervention: baseline | | Students |
| Delivering IcPAB intervention | Teachers | At IcPAB intervention venue | During the intervention period | 12 weeks | Students (Intervention Group) |
| Obtain mid-intervention measurements | Research Team | At school | During the mid-intervention: | Once | Students |
| Obtain end-intervention measurements | | | During the end-intervention: | | |
| Obtain information about the capability, the desire of the facilitators, and opportunities to implement an IcPAB intervention further | | At the field locations where | At the end of the intervention | Once from each key informant | Students and teachers |

| | | | | | |
|---|---|---|---|---|---|
| | | interviews are conducted | | | (Intervention Group) |
