# Supplementary material for: In-classroom physical activity breaks program among school children in Sri Lanka: study protocol for a randomized controlled trial
Source: Front Public Health. 2024 Apr 22;12:1360210. doi: 10.3389/fpubh.2024.1360210 (PMC11070516; doi:10.3389/fpubh.2024.1360210)
Supplement: Supplementary file 4 [file Data_Sheet_4.PDF]

Supplementary Table. Two selected IcPAB Cards as examples

<table>
<tr><th>IcPAB card with the instruction provided on its back</th><th>English translation of the instruction</th></tr>
<tr><td>
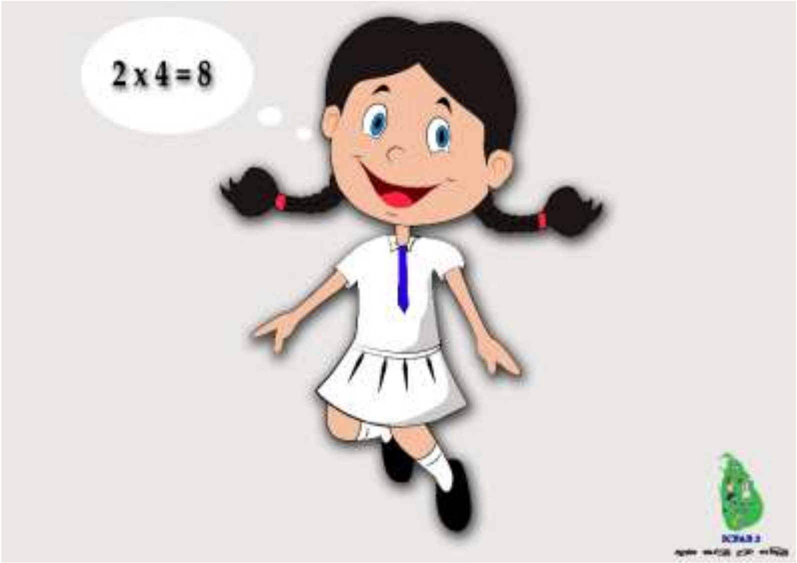

දරුවන්ට පහත සඳහන් උපදෙස් ලබා දෙන්න.

1. සුවපහසු ලෙස නැගී සිටින්න.
2. දෙපයේ ඇඟිලිවලින් ඉහළට එසවෙමින් (දෑත් ද එම රිද්මයටම ඉහළට ගෙන යන්න) සහ පහත්වෙමින් (දෑත් ද එම රිද්මයටම පහළට ගෙන යන්න) තෙවරක් හොඳින් ආශ්වාස-ප්‍රශ්වාස කරන්න.
3. ගුරුතුමා ලබාදෙන ගුණන ගැටලුව විසඳා (ගැටලුව වඩාත් සංකීර්ණ නම් කොළයක් මත විසඳා ගැනීමට යොමු කරන්න), ලැබෙන පිළිතුරෙහි සඳහන් ඉලක්කමට සමානවන පියවර ප්‍රමාණය එකතැන උඩපනිමින් ප්‍රදර්ශනය කරන්න.
4. නිවැරදි පිළිතුර ඔබ විසින් ද නැවත කළු ලෑල්ල මත විසඳන්න.

මෙම ක්‍රියාකාරකමෙහි අවම වශයෙන් මිනිත්තු පහක්වත් යෙදෙන්න.

ක්‍රියාකාරකම අවසන් කිරීම සඳහා ඇගයුම් වචන (බොහොම හොඳයි/ very good ආදී ලෙස) භාවිත කර, නැවත පාඩම වෙත යොමුවන්න.
</td><td>
Instruct the students to:

1. Stand comfortably.

2. Inhale-exhale three times by lifting up and down on the toes while moving the hands up and down in the same rhythm.

3. Solve the multiplication problem given by you /teacher (if the problem is more complicated, ask students to use a paper while standing), and to jump on the spot equal to the obtained answer.

4. Make sure to solve the question by yourself again on the blackboard.

Spend at least five minutes on this activity.

Use encouraging words (such as very good) to finish the activity and refer back to your lesson.
</td></tr>
</table>

<table>
<tr>
<td>
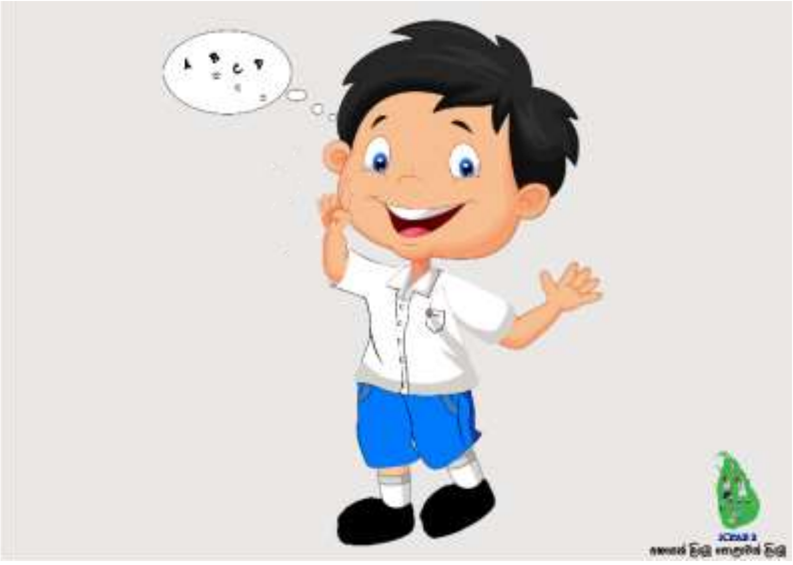

දරුවන්ට පහත සඳහන් උපදෙස් ලබා දෙන්න.

1. සුවපහසු ලෙස නැගී සිටින්න.
2. ගුරුතුමා පවසන අකුර හෝ වචනය ශබ්ද නගා කියවමින් අත භාවිත කර අවකාශයේ හෝ පාදය භාවිත කර පොළවෙහි ලියන්න.
3. නිවැරදි අක්ෂර වින්‍යාසය ඔබ විසින් නැවත කළු ලෑල්ල මත ලියා දරුවන්ට අදාළ ප්‍රතිපෝෂණය ලබා දෙන්න.

මෙම ක්‍රියාකාරකමෙහි අවම වශයෙන් මිනිත්තු පහක්වත් යෙදෙන්න.

ක්‍රියාකාරකම අවසන් කිරීම සඳහා ඇගයුම් වචන (බොහොම හොඳයි/ very good ආදී ලෙස) භාවිත කර, නැවත පාඩම වෙත යොමුවන්න.
</td>
<td>
Instruct the students to:

1. Stand comfortably.

2. Read aloud the letter or word the teacher says and write it using your hand in the space or your foot on the ground.

3. Make sure write each letter/ word on the blackboard with the correct spelling and give the children relevant feedback.

Spend at least five minutes on this activity.

Use encouraging words (such as very good) to finish the activity and refer back to your lesson.
</td>
</tr>
</table>
