# Supplementary material for: In-classroom physical activity breaks program among school children in Sri Lanka: study protocol for a randomized controlled trial
Source: Front Public Health. 2024 Apr 22;12:1360210. doi: 10.3389/fpubh.2024.1360210 (PMC11070516; doi:10.3389/fpubh.2024.1360210)
Supplement: Supplementary file 5 [file Data_Sheet_5.PDF]

Table: Synthesis of primary and secondary outcomes [1]

Level	Variable	Efficacy Endpoint	Instrument	Units
**Primary**	Academic achievement: **Mathematics**	Change in mathematics scores from baseline to 12 weeks	Standardized curriculum specific mathematics test designed by the teachers	Total marks obtained out of 100
	Academic achievement: **Reading**	Change in reading scores from baseline to 12 weeks	Standardized curriculum specific reading test designed by the teachers	Total marks obtained out of 100
**Secondary**	Physical activity levels	Change in length of time in light, moderate, vigorous, very vigorous and moderate-to-vigorous physical activity from baseline to 12 weeks during regular school hours	ActiGraph Gt3X+ and wGt3X-BT accelerometer	Length of time in light, moderate, vigorous, very vigorous and moderate-to-vigorous physical activity in minutes based on Evenson Child cut points
	Step counts	Change in step counts from baseline to 12 weeks during regular school hours		Sum of step counts based on Evenson Child cut points
	Sedentary behavior	Change in length of time in sedentary from baseline to 12 weeks during regular school hours		Length of time in sedentary based on Evenson Child cut points
	BMI	Change in BMI from baseline to 12 weeks	Anthropometry	Weight in kilograms and height in centimeters
	Aerobic fitness	Change in VO_2max from baseline to 12 weeks	20m shuttle run test	Weight in kilograms, height in centimeters, age in years, number of shuttles and levels completed
	Perceived stress	Change in perceived stress from baseline to 12 weeks	Perceived Stress Questionnaire 8–11 (PSS 8-11) [2]	Perceived stress rated on a four-point Likert scale from 'never' to 'vert often'

1. Ferreira da Silva dos Santos S, Bordin D, Dornelas de Souza EF, Freitas Júnior IF. Study protocol and baseline characteristics of "SCHOOL IN ACTION" program on support to physical activity and healthy lifestyles in adolescents. Contemp Clin Trials Commun. 2020;17:100505.

2. Snoeren F, Hoefnagels C. Measuring Perceived Social Support and Perceived Stress Among Primary School Children in The Netherlands. Child Indic Res. 2014;7:473–86.
