# Supplementary material for: In-classroom physical activity breaks program among school children in Sri Lanka: study protocol for a randomized controlled trial
Source: Front Public Health. 2024 Apr 22;12:1360210. doi: 10.3389/fpubh.2024.1360210 (PMC11070516; doi:10.3389/fpubh.2024.1360210)
Supplement: Supplementary file 6 [file Data_Sheet_6.PDF]

A screenshot of a log sheet

සතියේ දිනවල ඔබ විසින් IcPAB කාඩ්පතක් කොපමණ වාරයක් භාවිත කළාදැයි, IcPAB ක්‍රියාකාරකම් කේතය ඉදිරියෙන් ප්‍රගණන ලකුණු භාවිතෙයන් දක්වන්න.

සතිය → අගෝස්තු/ සැප්තැම්බර්/ ඔක්තෝම්බර් 2022/ 2023					
ක්‍රියාකාරකම් කේතය	සඳුදා	අඟහරුවාදා	බදාදා	බ්‍රහස්පතින්දා	සිකුරාදා
IcPAB 1					
IcPAB 2					
IcPAB 3					
IcPAB 4					
IcPAB 5					
IcPAB 6					
IcPAB 7					
IcPAB 8					
IcPAB 9					
IcPAB 10					
IcPAB 11					
IcPAB 12					
IcPAB 13					
IcPAB 14					
IcPAB 15					
IcPAB 16					
IcPAB 17					
IcPAB 18					
IcPAB 19					
IcPAB 20					
වෙනත් 1					
වෙනත් 2					

පන්තිභාර ගුරුතුමා/තුමියගේ අත්සන:

දිනය:

One log sheet is assigned to record the IcPAB implementation of a week.

The teacher should complete the log sheet everyday using tally numerations to indicate the number of IcPAB activities conducted.

More space is provided on the sheet to record teacher observations/ experience if necessary.
