# Supplementary material for: In-classroom physical activity breaks program among school children in Sri Lanka: study protocol for a randomized controlled trial
Source: Front Public Health. 2024 Apr 22;12:1360210. doi: 10.3389/fpubh.2024.1360210 (PMC11070516; doi:10.3389/fpubh.2024.1360210)
Supplement: Supplementary file 7 [file Data_Sheet_7.PDF]

Process evaluation questionnaires

PROCESS EVALUATION QUESTIONNAIRE FOR TEACHERS

(English Version)

Read the statements in the table below and draw a cross over the face/ emoji that you think is most applicable to you to indicate your answer.

😩	🙁	😐	🙂	😃
Not agree at all				Very much agree

The classroom-based physical activity breaks (IcPAB):					
1. fitted well into the daily school routine	😩	🙁	😐	🙂	😃
2. was easy to adapt to my class	😩	🙁	😐	🙂	😃
3. created many additional works	😩	🙁	😐	🙂	😃
4. was not flexible at all	😩	🙁	😐	🙂	😃
5. contained much to be done	😩	🙁	😐	🙂	😃
6. allotted too long at a time	😩	🙁	😐	🙂	😃
7. will be used by me in future too	😩	🙁	😐	🙂	😃
8. will be recommended to other teachers	😩	🙁	😐	🙂	😃

PROCESS EVALUATION QUESTIONNAIRE FOR STUDENTS

(English Version)

Daughter/ Son, Read the statements in the table below and draw a cross over the face/ emoji that you think is most applicable to you to indicate your answer.

😩	😟	😐	🙂	😃
Not Agree at All				Very Much Agree

**IcPAB (Classroom-based Physical Activity Breaks):**					
1. I happily engaged in the activities	😩	😟	😐	🙂	😃
2. I like to do in future too	😩	😟	😐	🙂	😃
3. I felt happy compared to before	😩	😟	😐	🙂	😃
4. I could easily do them	😩	😟	😐	🙂	😃
5. I could easily understand them	😩	😟	😐	🙂	😃
6. I had enough space near the table	😩	😟	😐	🙂	😃
7. I felt that the allocated time was enough	😩	😟	😐	🙂	😃
8. I did enough activities each day	😩	😟	😐	🙂	😃
9. The speed I did the activities was enough	😩	😟	😐	🙂	😃

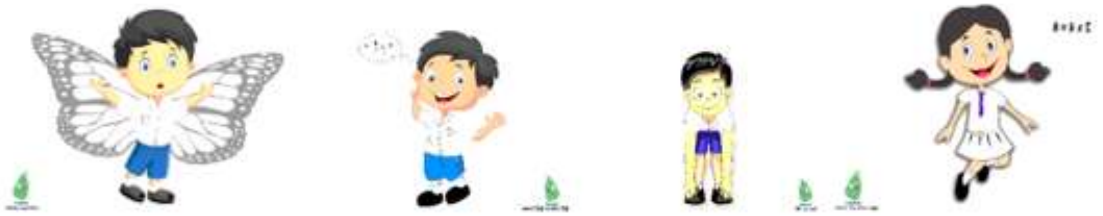

Name - ....

Class - ....
