# Supplementary material for: In-classroom physical activity breaks program among school children in Sri Lanka: study protocol for a randomized controlled trial
Source: Front Public Health. 2024 Apr 22;12:1360210. doi: 10.3389/fpubh.2024.1360210 (PMC11070516; doi:10.3389/fpubh.2024.1360210)
Supplement: Supplementary file 8 [file Data_Sheet_8.PDF]

Post Intervention: INTERVIEW GUIDE – FOR TEACHERS

(English Version)

**Introduction**<br><br>**Opening Statement and Warm-up**	Thank you very much for informing me of your consent to participate in this interview. I would like to ask some questions about the physical activity breaks/ IcPAB that you implemented for 12 weeks in your classroom. Any opinion that you would share is highly appreciated, as that will be helpful for our research team to introduce an activity-based intervention for school kids. To make sure that I do not miss the data that you provide, would you like me to record our conversation? Of course, this conversation is confidential and, I ensure your privacy. Furthermore, I am using your responses only for research-related purposes. I will permanently delete all the primary data that you have provided within one year, after completing this study. If you agree, may I ask you to verbally mention that you agree with the interview setting that I have explained to you now? Please feel free to ask any questions if you have any at this stage.	
**Transitioning to...**	**Question(s)**	**Probe(s)**
Approaching towards the interview topic with a convenient talk related to IcPAB	So, please tell me in general, how was your overall experience with this IcPAB programme?	Were you always happy to implement IcPAB, or were you annoyed by this programme?
**Main body (Topic I to**	Now, I would like to concentrate more on your perceptions regarding	

<table>
<tr><td>IV)</td><td colspan="2">the IcPAB programme. But, if you like to share any perceptions in addition to my specific questions, you are always welcome to speak 😊.</td></tr>
<tr><td>Topic I

Outcome Evaluation</td><td colspan="2">1. Can you explain how the students' mathematics and reading achievement were impacted by our IcPAB programme?
2. Could you please provide me any example if you have witnessed improved levels of physical activities and walking or decreased levels of seated learning after the IcPAB was implemented?
3. Do you think that the BMI or aerobic fitness of the students improved during past three months? Why do you say so?
4. Can you elaborate any witnessing of stress among the students during the intervention period compared to the previous school term?</td></tr>
<tr><td rowspan="4">Topic II

Capabilities

(Capability is defined as the individual's psychological and physical capacity to engage in the activity concerned.)</td><td>1. Please tell me, were you able to implement all the IcPAB activities within the 12 weeks of time?</td><td>Can you explain to me more on how and how often you have done those?</td></tr>
<tr><td>2. Did you receive enough knowledge and skills to implement the IcPAB activities?</td><td>Or do you require more support in terms of knowledge and skills to do the classroom-based physical activity breaks?</td></tr>
<tr><td>3. Did you find any IcPAB card that made it difficult for you to implement? If yes, please elaborate them.</td><td></td></tr>
<tr><td>4. Do you think that you were physically okay to implement the IcPAB activities?</td><td>Were there any activities that were hard to implement due to any of your physiological limits?</td></tr>
</table>

	5. Were you able to manage doing activities within 5 minutes each time? Please explain.	
	6. How about your psychological readiness to implement the activities?	Were there any circumstances that lured your mind to not to do any activity(ies)?
	Alright. Let's think about the factors that influenced you (I mean factors that lie outside you 😊) during the implementation of this 12-week IcPAB programme.	
**Topic III**<br>**Opportunities**<br>(Opportunity is defined as all the factors that lie outside the individual that make the behaviour possible or prompt it)	7. What do you think about the opportunities to carry out the physical activity breaks inside the classroom? If you found some opportunities, please elaborate them. /Did you receive enough support from your peer teachers, section heads, principal and the school as whole to be involved in and engage in this programme?	Can you give me some examples?
	8. Did you receive enough opportunities to adapt the IcPAB activities to be fitted with the classroom's workload? Or did you find enough opportunities to carry out the physical activity breaks inside the classroom?	May you share some experiences on that aspect with me?
	9. Could you please tell me whether you were asked to stop or carry on the IcPAB activities by anybody by giving any reason or without giving a reason?	In what ways or how were the outsiders gave opportunities to make the IcPAB happen?
	10. How would you assess the support that you received from the University's team in continuing the IcPAB activities?	Let's say 4 is very supportive, 3 is supportive,

		2 seldomly supportive and<br>1 is not supportive at all…
	11. Do you think that you have received enough opportunities from our team to ask questions, obtain clarifications regarding the IcPAB activities?	Can you tell me why do you think so?
	12. How many activity breaks could you implement in a regular school day? Why?	Did you have enough opportunities to implement the IcPAB activities at least three times per day? Why?
**Topic IV**<br>**Motivations**<br>(Motivation is defined as all those brain processes that energize and direct behaviour, not just goals and conscious decision-making)	Okay! Let's discuss about your own motivation in implementing the IcPAB.	
	13. You were asked to implement the IcPAB activities at least three times per day. Were you energised to do so? Or did you feel like not reaching towards that goal?	Could you elaborate, the reasons, please?
	14. Every day, our team sent you a WhatsApp message to remind you of our daily goal. Even before receiving that message, have you ever started initiating an activity break daily?	When WhatsApp reminders were not sent to you on some days, were you self-motivated to do the IcPAB? If not, please explain why you were unmotivated to do the IcPAB?
	15. Have you ever felt that you are also enjoying the IcPAB, and you are going to continue these activities even after the 12 weeks of time?	What made your mind to motivate/ demotivate you

		that way?
	16. Did you face any barriers that hindered your interest to implement IcPAB activities?	Can you please share any experience on that with me?
	17. Based on your experience do you think that you need more motivations to implement the activity breaks daily? In what ways?	
**Cool-off**	Okay. In addition to what I have asked, do you have anything more to share with me? Anything which points out the strengths or weaknesses of this programme? Or any changes that you would like to see in the introduced IcPAB?	
**Closure**	Great! This is the end of our interview, then. Thank you very much for your enormous support and time!	

Post Intervention: INTERVIEW GUIDE – FOR STUDENTS

(English Version)

**Introduction**<br><br>**Opening Statement**	Thank you very much for informing me of your consent to participate in this interview. I would like to ask some questions about the physical activity breaks/ IcPAB that you did with your teachers in your classroom. Any opinion that you would share is highly appreciated. Anyone of you can speak even though you all are sitting together herewith me. To make sure that I do not miss the data that you provide, would you like me to record our conversation? Of course, this conversation is confidential and, I ensure your privacy. Furthermore, I am using your responses only for the research-related purposes. I will permanently delete all the primary data that you have provided within one year, after completing this study. If you agree, may I ask you to verbally mention that you agree with the interview setting that I have explained to you now? Please feel free to ask any question if you have any at this stage.	
**Transitioning to...**	**Question(s)**	**Probe(s)**
**Warm-up**	First of all, would you like to share something about yourself with me?	And how do you feel today?
	Thank you very much for sharing your information with me.	
Approaching towards the	So, please tell me in general,	Were you always happy to do

interview topic with a convenient talk related to IcPAB	did you enjoy the IcPAB?	the IcPAB when your teacher asked you to do so, or were you annoyed by this programme?
	Which activities did you like the most? And which activities did you hate the most?	
**Main body (Topic I to IV)**	Now, I would like to more about how the teacher did the activities with you. But, if you like to share any perceptions in addition to my specific questions, you are always welcome to speak 😊.	
**Topic I**<br>Outcome Evaluation	1. Did you feel that your mathematics abilities improved a lot after engaging in the IcPAB with your teachers? Why do you say so?<br>2. Did you feel that your reading abilities improved a lot after engaging in the IcPAB with your teachers? Why do you say so?<br>3. Try to remind yourself before you did the IcPAB and after you did the IcPAB. How would you explain your fitness?<br>4. Try to remind yourself before you did the IcPAB and after you did the IcPAB. How would you explain your stress, fears, or happiness? That can be related to your studies or scholarship exam or anything…	
**Topic II**<br>Capabilities<br>(Capability is defined as the individual's psychological and physical capacity to	1. Did you enjoy the pictures shown on IcPAB cards?<br>2. Did your teacher clearly explain the IcPAB to you?	Do you think that the teacher could clearly provide you the instruction to do the IcPAB activities?
	3. Were you capable to follow teacher's instructions?	Was there any activity which

engage in the activity concerned.)		was difficult for you to follow? If yes, please tell me what they are?
	4. Were there any circumstances that made your mind to not to do IcPAB activity(ies) when your teacher asked you to do so?	Can you share any incidence with me?
	Alright. Let's think about the time that you spent on doing activity breaks.	
	5. What do you think about the chances to participate in IcPAB? If you recall some chances, please tell me what they are?	Why do you think so? / If you recall some chances, please tell me what they are?
**Topic III** Opportunities (Opportunity is defined as all the factors that lie outside the individual that make the behaviour possible or prompt it)	6. When you were tired or felt like not learning did your teacher do any IcPAB with you?	
	7. Did you enjoy Hashi aunty and her friends' company when you were asked about the IcPAB each week?	Why do you say so?
	8. How many activity breaks did you do in last (the last school day before the interview date)?	
**Topic IV** Motivations (Motivation is defined as all those brain processes that	Okay! Let's discuss about your own motivation in doing the IcPAB.	
	9. Have you ever suggested the teacher to do an IcPAB when you felt bored?	In what ways did your teacher respond to you when you feel

energize and direct behaviour, not just goals and conscious decision-making)		tired or bored?
	10. Did you feel like not doing the IcPAB during the past days? If yes, please tell me why you were unmotivated to do the IcPAB?	Can you explain why?
	11. Do you prefer your teacher to do these activities in future, too?	Why?
**Cool-off**	Okay. In addition to what I have asked, do you have anything more to share with me? Anything that you would like to share about the introduced IcPAB?	
**Closure**	Great! This is the end of our interview, then. Thank you very much for your time!	
